# Glycogen synthase kinase 3ß functions as a positive effector in the WNK signaling pathway

**DOI:** 10.1371/journal.pone.0193204

**Published:** 2018-03-01

**Authors:** Atsushi Sato, Hiroshi Shibuya

**Affiliations:** Department of Molecular Cell Biology and Joint Usage/Research Center for Intractable Diseases, Medical Research Institute, Tokyo Medical and Dental University (TMDU), Bunkyo-ku, Tokyo, Japan; Rutgers University, UNITED STATES

## Abstract

The with no lysine (WNK) protein kinase family is conserved among many species. Some mutations in human WNK gene are associated with pseudohypoaldosteronism type II, a form of hypertension, and hereditary sensory and autonomic neuropathy type 2A. In kidney, WNK regulates the activity of STE20/SPS1-related, proline alanine-rich kinase and/or oxidative-stress responsive 1, which in turn regulate ion co-transporters. The misregulation of this pathway is involved in the pathogenesis of pseudohypoaldosteronism type II. In the neural system, WNK is involved in the specification of the cholinergic neuron, but the pathogenesis of hereditary sensory and autonomic neuropathy type 2A is still unknown. To better understand the WNK pathway, we isolated WNK-associated genes using *Drosophila*. We identified Glycogen synthase kinase 3ß (GSK3ß)/Shaggy (Sgg) as a candidate gene that was shown to interact with the WNK signaling pathway in both *Drosophila* and mammalian cells. Furthermore, GSK3ß was involved in neural specification downstream of WNK. These results suggest that GSK3ß/Sgg functions as a positive effector in the WNK signaling pathway.

## Introduction

The with no lysine (WNK) protein kinases are atypical members of the serine/threonine kinase family, and are conserved among many species [1–3]. The mammalian WNK family has four members: WNK1–4. *WNK1* and *WNK4* have been identified as causative genes of pseudohypoaldosteronism type II (PHAII) [4], and *WNK1* is also a causative gene of hereditary sensory and autonomic neuropathy type 2A (HSAN2A) [5]. Several groups including ours have attempted to identify the functions of the WNK family. In the kidney, WNK1 and WNK4 phosphorylate and activate STE20/SPS1-related, proline alanine-rich kinase (SPAK) and oxidative-stress responsive 1 (OSR1) kinases, which in turn regulate various ion co-transporters [6–9]. Because knock-in mice of *Wnk4*^*D561A*^ (the mutation found in PHAII patients) display similar phenotypes to PHAII, dysregulation of this WNK signaling pathway was thought to cause hypertension in PHAII patients [10]. In the neural system, a neural-specific alternatively spliced isoform of *WNK1* is expressed, which includes the neural-specific exon *HSN2*. In HSAN2A patients, mutations were found in this *HSN2* exon [5, 11], but *HSN2* knock-out mice have no discernable morphological phenotype [11, 12]. Furthermore, in other familial HSAN2A patients, mutations were found outside the *HSN2* exon in *WNK1* [13]. Thus, the pathogenesis of HSAN2A remains unclear.

WNK kinases are required for EGF-mediated ERK5 activation, and WNK family members are also involved in proliferation, migration, and differentiation [14–16]. Recently, we found that WNK1 and WNK4 induced *Lhx8* expression and were important for neural specification [17]. Moreover, WNK was identified as a positive regulator of the Wnt signaling pathway; however, the detailed mechanisms of this are unknown [18]. Although WNK has a range of functions during many developmental processes, little is known about the components of the WNK signaling pathway, except for the main molecules WNK1/4–SPAK/OSR1. In the kidney, ASK3 inhibits WNK1 [19], and the PI3K/AKT signaling pathway activates the WNK–SPAK/OSR1–NCC pathway [20]. Other upstream or downstream component(s) are still unknown.

Glycogen synthase kinase 3ß (GSK3ß) is a ubiquitously expressed serine/threonine kinase that was originally identified as the regulatory kinase of glycogen synthase. Since then, GSK3ß has been shown to be involved in many biological processes [21]. GSK3ß plays important roles in several signaling pathways, especially PI3K/AKT and Wnt signaling pathways. In the PI3K/AKT signaling pathway, AKT phosphorylates Ser9 of GSK3ß which inhibits its activity, thus phosphorylating cyclin D1 and regulating the cell cycle [22]. In the Wnt signaling pathway, GSK3ß is a major component of the destruction complex that phosphorylates ß-catenin, which in turn is degraded by proteasomes [22].

In this study, we attempted to identify a new component of the WNK signaling pathway using *Drosophila*, and identified the *shaggy* gene (*sgg*) as a possible candidate. Sgg is a *Drosophila* homolog of mammalian GSK3ß. We found that GSK3ß worked as a positive effector downstream of WNK in both mammalian and *Drosophila* cells.

## Materials and methods

### Ethics statement

All the animal experiments were performed under the ethical guidelines of Tokyo Medical and Dental University, and animal protocols were reviewed and approved by the animal welfare committee of the Tokyo Medical and Dental University.

### Fly stocks and genetics

Fly strains used in this study were; Canton-S, *y w*, EY10165 (UAS-*Wnk*; Bloomington Stock Center #16970), UAS-*fray*^*S347D*^ [17], *Wnk*^*EY18*^ FRT2A [17], *Akt1*^*04226*^ (Bloomington Stock Center #11627), *sgg*^*1*^ (Bloomington Stock Center #9095), *sgg*^*M11*^ (Bloomington Stock Center #31308), UAS-*sgg* (Bloomington Stock Center #5435), *hh-Gal4* (Bloomington Stock Center #67046), *arm*-*Gal4* (Bloomington Stock Center #1560), *hsGFP hsCD2*(*y*^*+*^) *M(3)i55 Tub>Gal80* FRT2A (provided by G. Struhl).

We made *hh*-*Gal4* EY10165 recombinant flies for screening. We crossed these flies with the *fray* mutant and confirmed the suppression as described previously [17]. For initial screening, we crossed several mutants and isolated candidate suppressor genes (data not shown), including *sgg*.

### Histology and staining

All wings were mounted in GMM [23]. Images were obtained using SteREO Discovery and Axioscope microscopes (Carl Zeiss), and were processed using Axiovision with extended focus (Carl Zeiss) and Photoshop (Adobe).

### Cultured cell lines

Neuro2A cells [17] were grown in DMEM with 20% FBS. Polyethylenimine (Polysciences) was used to transfect plasmids, and the *Trans*IT-X2 Dynamic Delivery System (Mirus Bio) was used to transfect small interfering (si)RNA and co-transfect siRNA and plasmids. The plasmids we used are: pRK5-Flag-hWNK1, pRK5-Flag-hWNK1^D368A^, pRK5-Flag-OSR1, pRK5-T7-OSR1, pRK5-Flag-OSR1^K46M^, pRK5-T7-OSR1^K46M^, pRK5-Flag-OSR1^S325D^, pcDNA-Flag-GSK3ß, pRK5-Flag-GSK3ß, pRK5-Flag-GSK3ß^K85M^. siRNA target sequences were described previously [17], and were as follows: mouse Osr1, 5′-GAUAUUCGAUUUGAAUUUA-3′; and mouse *GSK3ß*, 5′-GAAAUGAACCCAAAUUAUA -3′. To differentiate Neuro2A cells, they were induced in serum-free DMEM with 10 μM retinoic acid for 24 h.

### Immunoprecipitation

Neruo2A cells were transfected with indicated expression vectors. Then, lysates were prepared from transfected cells, and immunoprecipitated with indicated antibodies and Protein A/G PLUS-agarose (Santa Cruz). Immunoprecipitates were subjected to SDS-PAGE and western blotting, and bands were detected by the LAS-4000 mini (GE) image analyzer. For sequential immunoprecipitation, the anti-Flag antibody (M2, Sigma) was used and eluted with Flag peptides (Sigma). Eluates were divided and immunoprecipitated with anti-T7 antibodies and control mouse normal IgG.

### RT-PCR analysis

Total RNA was isolated by TRIzol (Invitrogen). cDNA synthesis was carried out using Moloney murine leukemia virus reverse transcriptase (Invitrogen). *GAPDH* was used for the normalization of cDNA samples. Primer pairs were described previously [17], and were as follows: mouse *GSK3ß*, 5′-GCAGCAAGGTAACCACAGTAGTGGC-3′ and 5′-TGGTGCCCTGTAGTACCGAGAACAG-3′.

### *In vitro* kinase assay

Neuro2A cells were transfected with Flag-WNK1, Flag-WNK1^D368A^, Flag-OSR1, Flag-OSR1^K46M^, or Flag-OSR1^S325D^ expression plasmids. Lysates were prepared from transfected cells and immunoprecipitated with an anti-Flag M2 antibody (Sigma) and Protein A/G PLUS-agarose (Santa Cruz). Immunoprecipitates were incubated with bacterially-expressed GST fusion proteins (GST-GSK3ß ^K85M^) in kinase buffer containing 10 mM HEPES (pH 7.4), 1 mM DTT, 5 mM MgCl_2_, and 5 μCi [γ-^32^P]-ATP at 30°C. Phosphorylated substrates were subjected to SDS-PAGE, and bands were detected by the FLA3000 image analyzer (Fujifilm).

### Antibodies

Antibodies used in this report were: mouse anti-Flag M2 (Sigma; 1:400 for immunoprecipitation), rabbit anti-Flag (Sigma; 1:1000 for western blotting), rat anti-HA (Roche; 1:1000 for western blotting), mouse anti-T7 (Merck; 1:2000 for immunoprecipitation), rabbit anti-T7 (MBL; 1:1000 for western blotting), anti-rabbit HRP-conjugated (GE; 1:10000 for western blotting), and anti-rat HRP-conjugated (GE; 1:10000 for western blotting).

### Quantification and statistical analysis

Quantitative PCR was performed with an Applied Biosystems 7300 Real-Time PCR Cycler (ABI) using THUNDERBIRD SYBR qPCR Mix (TOYOBO). Primer sequences for *Lhx8*, *ChAT*, *Gad1*, and *GAPDH* were described previously [17]. *GAPDH* was used for the normalization of cDNA samples. Neurite lengths were measured using ImageJ software (NIH). Data were computed using Microsoft Excel (Microsoft) and StatPlus (AnalystSoft). Values and error bars represent the means and SDs, and are representative of at least three independent experiments.

## Results

### Shaggy is a novel candidate effector of the WNK signaling pathway

**Fig 1:**
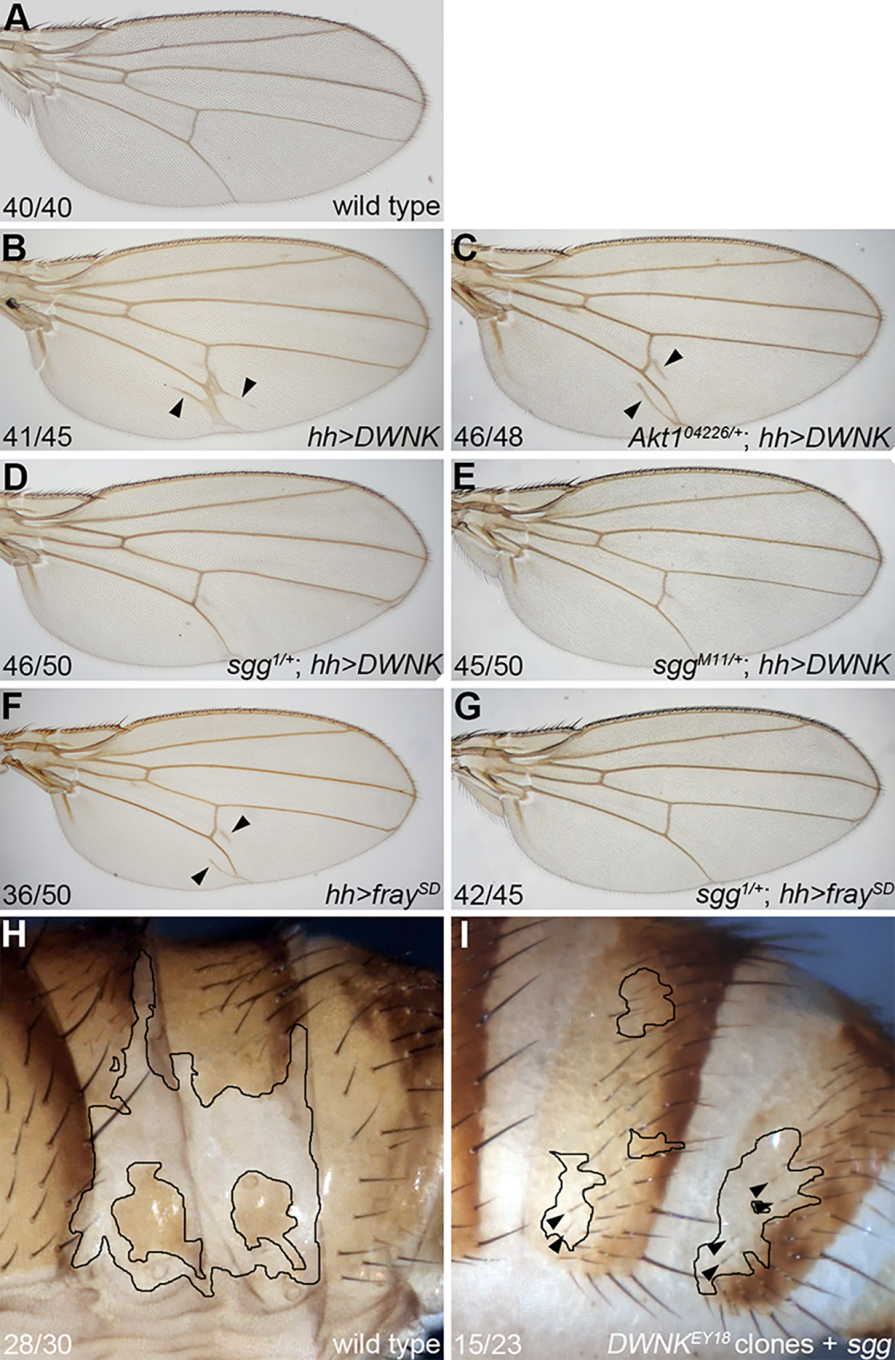
*sgg* is downstream of *Wnk* in the *Drosophila* wing vein and abdominal patterning. (A) Wild-type wing. (B) Wing from EY10165 (UAS-*Wnk*) fly driven by *hh*-*Gal4*. Additional veins around vein 5 (arrowhead) were observed. (C) Wing from fly overexpressing *Wnk* driven by *hh*-*Gal4* with the *Akt1*^*04226*^ heterozygous mutant. (D) Wing from fly overexpressing *Wnk* driven by *hh*-*Gal4* with the *sgg*^*1*^ heterozygous mutant. (E) Wing from fly overexpressing *Wnk* driven by *hh*-*Gal4* with the *sgg*^*M11*^ heterozygous mutant. (F) Wing from UAS-*fray*^*S347D*^ fly driven by *hh*-*Gal4*. Additional veins around vein 5 (arrowhead) were observed. (G) Wing from fly overexpressing *fray*^*S347D*^ driven by *hh*-*Gal4* with the *sgg*^*1*^ heterozygous mutant. (H) Abdomen from adult fly with *DWNK*^*EY18*^ minute clones. Thin black lines indicate the clone border. (I) Abdomen from adult fly with *DWNK*^*EY18*^ minute clones and *sgg* overexpression. *sgg* was expressed only in *DWNK*^*EY18*^ minute clones using the *Gal80* suppression technique. Thin black lines indicate the clone border (also the *sgg* expression area). Black arrowheads show rescued abdominal bristles. The detailed genotype is *y w hsflp*; *arm*-*Gal4* / UAS-*sgg*; *Wnk*^*EY18*^ FRT2A / *hsGFP hsCD2*(*y*^*+*^) *M(3)i55 Tub>Gal80* FRT2A. The numbers of wings or abdomina showing the phenotypes and of total observed wings or abdomina are indicated.

Overexpression of *Drosophila WNK* using *hh-Gal4* driver resulted in an ectopic vein around vein 5 in the adult wing (Fig 1B compared with Fig 1A; [17]). As we showed previously, a heterozygous mutation of *fray*, which encodes a *Drosophila* homolog of SPAK/OSR1 that is a downstream effector of WNK, suppressed this phenotype [17]. Therefore, we performed screening to identify a new effector of the WNK signaling pathway using this system. We selected several mutants known to be components of other signaling pathways, such as the Wnt pathway, Notch pathway, TGFß pathway, and the EGF pathway. We obtained several candidate suppressor genes, including *sgg*, which encodes the *Drosophila* homolog of mammalian GSK3ß whereas some genes, including *Akt1*, did not show any interaction (Fig 1C and data not shown). Two independent *sgg* mutants (*sgg*^*1*^ and *sgg*^*M11*^) suppressed the wing phenotypes by the overexpression of *Wnk* (Fig 1D and 1E), suggesting that *sgg* is a suppressor of the WNK signaling pathway and that this suppression is not an effect of the genetic background.

We further tested the interaction between *sgg* and *fray*. Ectopic expression of *fray*^*S347D*^ (the constitutively active form of *fray*) resulted in a similar phenotype to that seen following the ectopic expression of *Wnk* (Fig 1F; [17]). The *sgg*^*1*^ mutant also repressed these phenotypes (Fig 1G) suggesting that *sgg* interacts with the WNK signaling pathway in *Drosophila*.

We next confirmed this genetic interaction between *Wnk* and *sgg*. Because *Wnk*^*EY18*^ mutant clones led to abdominal developmental defects (Fig 1H; [17]), we attempted to rescue this phenotype by the overexpression of *sgg*. Using a combination of the FLP/FRT mosaic system and Gal80 suppression, we induced the local expression of *sgg* in *Wnk*^*EY18*^ minute clones. As shown in Fig 1I, *sgg* overexpression partially rescued the abdominal phenotype of the *Wnk*^*EY18*^ minute clones. These results suggest that *sgg* is a novel effector of the WNK signaling pathway, not only in wing development but also in abdominal development.

### GSK3ß functions as a positive effector downstream of WNK

**Fig 2:**
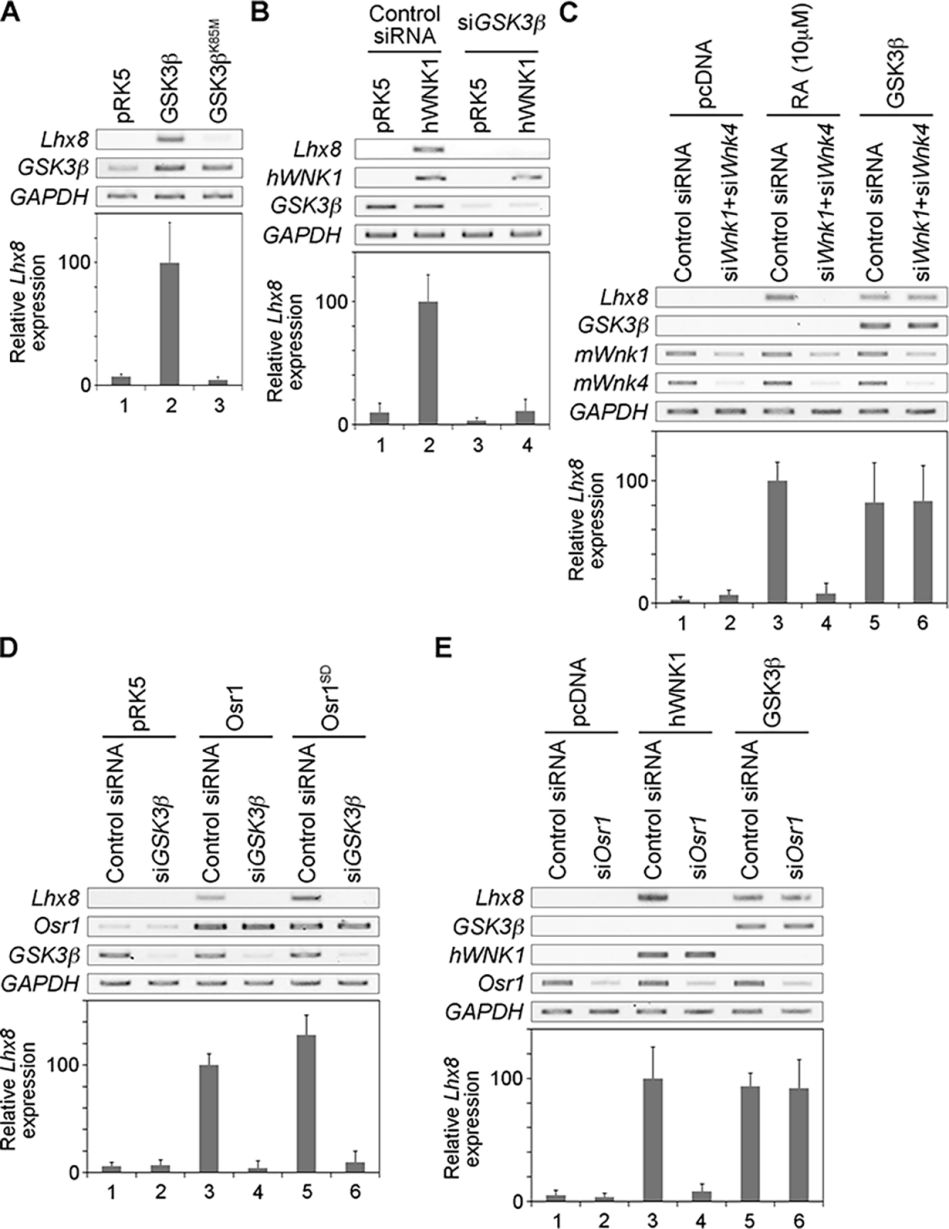
GSK3ß is a positive effector downstream of WNK-OSR1. (A) Gene expression determined by RT-PCR or quantitative RT-PCR analysis was examined in Neuro2A cells overexpressing GSK3ß or GSK3ß ^K85M^. The value obtained from each sample was normalized to that of *GAPDH*. The value of *Lhx8* from overexpressing GSK3ß (lane 2) was set to 100. (B) Gene expression detemined by RT-PCR or quantitative RT-PCR analysis was examined in Neuro2A cells overexpressing hWNK1 under *GSK3ß* knockdown using siRNA. The value obtained from each sample was normalized to that *GAPDH*. The value of *Lhx8* from overexpressing hWNK1 (lane 2) was set to 100. (C) Gene expression determined by RT-PCR or quantitative RT-PCR analysis was examined in Neuro2A cells stimulated by retinoic acid (RA) or overexpressing GSK3ß under both *Wnk1* and *Wnk4* knockdown using siRNA. The value obtained from each sample was normalized to that of *GAPDH*. The value of *Lhx8* from overexpressing GSK3ß (lane 3) was set to 100. (D) Gene expression determined by RT-PCR or quantitative RT-PCR analysis was examined in Neuro2A cells overexpressing OSR1 or OSR1^S324D^ (constitutively active form of OSR1) under *GSK3ß* knockdown using siRNA. The value obtained from each sample was normalized to that of *GAPDH*. The value of *Lhx8* from overexpressing OSR1 (lane 3) was set to 100. (E) Gene expression determined by RT-PCR or quantitative RT-PCR analysis was examined in Neuro2A cells overexpressing hWNK1 or GSK3ß under *Osr1* knockdown using siRNA. The value obtained from each sample was normalized to that of *GAPDH*. The value of *Lhx8* from overexpressing hWNK1 (lane 3) was set to 100.

Because *GSK3ß* and the WNK pathway are highly conserved among many species [2, 3, 22], we next examined whether the interaction between WNK and GSK3ß was also conserved in mammalian cells. In Neuro2A cells, WNK1 expression induced the expression of *Lhx8* ([17]; see also Fig 2B lane 2). As shown in Fig 2, the expression of GSK3ß also induced the expression of *Lhx8* in Neuro2A cells (Fig 2A lane 2). However, the kinase dead form of GSK3ß (GSK3ß ^K85M^) could not activate the expression of *Lhx8*, suggesting that GSK3ß kinase activity is required for its expression (Fig 2A lane 3).

Next, we examined the epistatic interaction between WNK1 and GSK3ß. The induction of *Lhx8* was suppressed by the knockdown of *GSK3ß* (Fig 2B lane 4). However, *Lhx8* induction by GSK3ß was not suppressed by the knockdown of both *Wnk1* and *Wnk4* (Fig 2C lanes 5 and 6), even though this knockdown did suppress *Lhx8* induction by retinoic acid (RA) stimulation (Fig 2C lanes 3 and 4).

The expression of OSR1, a downstream molecule of WNK, and its constitutively active form (OSR1^S325D^) induced the expression of *Lhx8* (Fig 2D lanes 3 and 5; [17]). This activation was also suppressed by the knockdown of *GSK3ß* (Fig 2D lanes 4 and 6). In contrast, the induction of *Lhx8* by GSK3ß was not suppressed by the knockdown of *Osr1* (Fig 2E lanes 5 and 6), although the induction of *Lhx8* by WNK1 was suppressed by the knockdown of *Osr1* (Fig 2E lanes 3 and 4). These data suggest that the WNK–OSR1–GSK3ß pathway is conserved not only in flies but also in mammals, and that GSK3ß functions as a positive effector downstream of the WNK signaling pathway.

### WNK1 and OSR1 form a complex with GSK3ß

**Fig 3:**
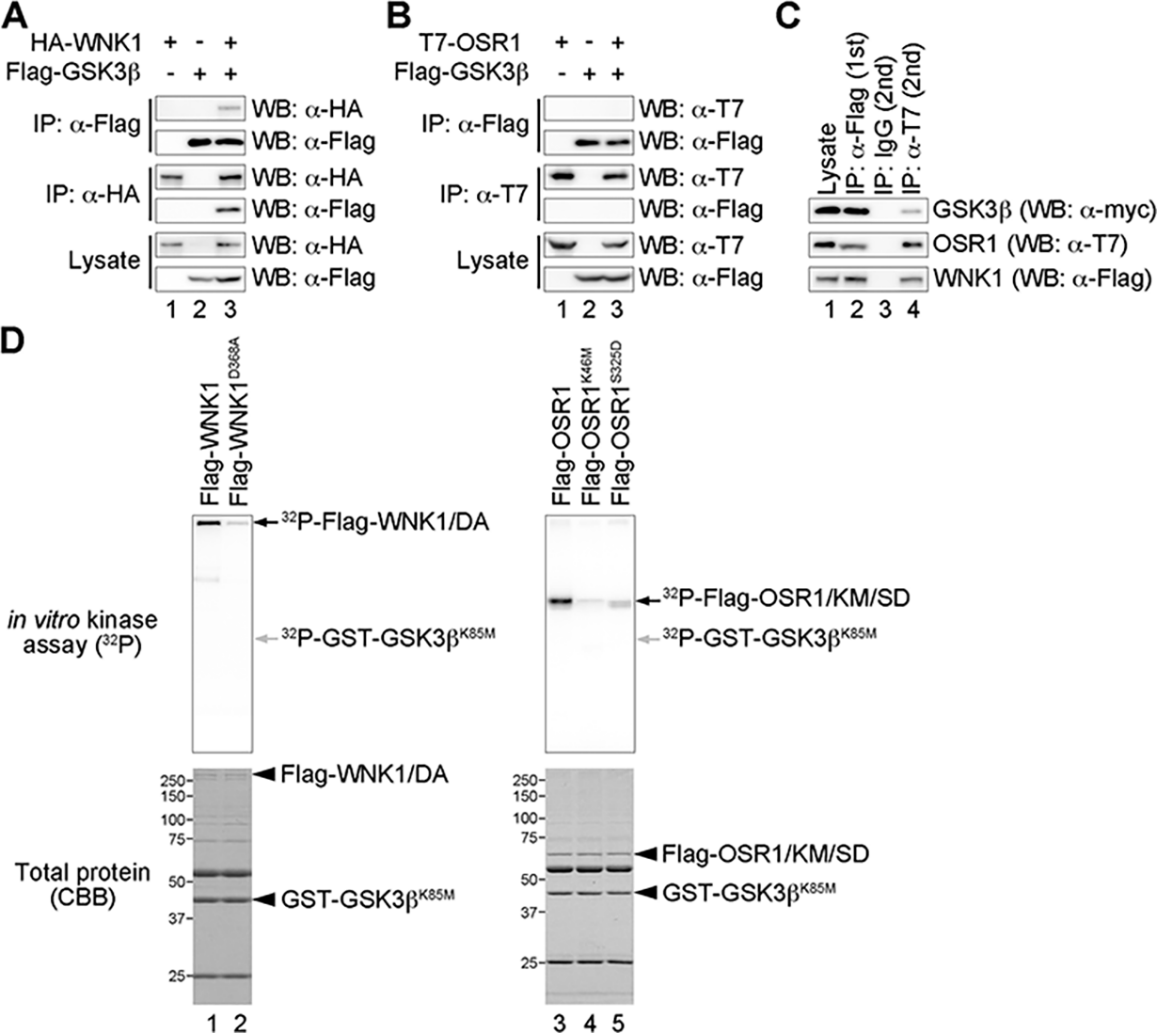
GSK3ß forms a complex with WNK1, but is not directly phosphorylated. (A–B) Interactions between WNK1 (A) or OSR1 (B), and GSK3ß were examined in Neuro2A cells by co-immunoprecipitation. Immunoprecipitates (IP) were subjected to western blotting (WB) with the indicated antibodies. +, present; -, absent. (C) Interaction among WNK1, OSR1 and GSK3ß were examined in Neuro2A cells by sequential immunoprecipitation. An anti-FLAG antibody was used for the first immunoprecipitation, and immunoprecipitates were subjected to a second immunoprecipitation with IgG or T7 antibodies. (D) Phosphorylation of GSK3ß by WNK1 or OSR1. Upper panel shows the result of an *in vitro* kinase assay. Black arrow in the left upper panel (^32^P-Flag-WNK1/DA) represents the auto-phosphorylation of WNK1. Black arrow in the right upper panel (^32^P-Flag-OSR1/KM/SD) represents the auto-phosphorylation of OSR1. Grey arrows in both left and right upper panels indicated the size of GSK3ß (^32^P-GST-GSK3ß ^K85M^). Lower panels show the total protein. Arrowheads in both left and right lower panels represent the indicated proteins.

We next investigated the biochemical interaction between WNK1 and GSK3ß. We transiently expressed Flag-tagged GSK3ß together with HA-tagged WNK1 or T7-tagged OSR1. When cell extracts were subjected to immunoprecipitation with anti-Flag, anti-HA (for WNK1), or anti-T7 (for OSR1) antibodies followed by immunoblotting, we found that GSK3ß interacted with WNK1 (Fig 3A lane 3), but not with OSR1 (Fig 3B lane 3). Because WNK1 bound to OSR1 [6, 7], we investigated whether the WNK1–OSR1 complex interacted with GSK3ß. As shown in Fig 3C, GSK3ß was immunoprecipitated after sequential IP by anti-Flag and anti-T7 antibodies, suggesting that GSK3ß forms a complex with WNK1 and OSR1.

GSK3ß is positively and negatively regulated by phosphorylation [24]. Because WNK1 and OSR1 are both Ser/Thr kinases [3, 25, 26], and WNK1–OSR1–GSK3ß forms the complex shown above, we examined whether WNK1 or OSR1 directly phosphorylated and regulated GSK3ß. To perform the *in vitro* kinase assay, we purified Flag-tagged WNK1 or OSR1 from cultured cell extracts, and produced a GST-fusion protein of the kinase dead form of GSK3ß (GST-GSK3ß ^K85M^) in bacteria. We did not observe phosphorylation of GSK3ß by WNK1 or OSR1 (Fig 3D lanes 1–5). These results suggest that GSK3ß forms a complex with WNK1 and OSR1, but that the regulation of GSK3ß by the WNK signaling pathway does not depend on direct phosphorylation.

### GSK3ß is involved in neural specification

**Fig 4:**
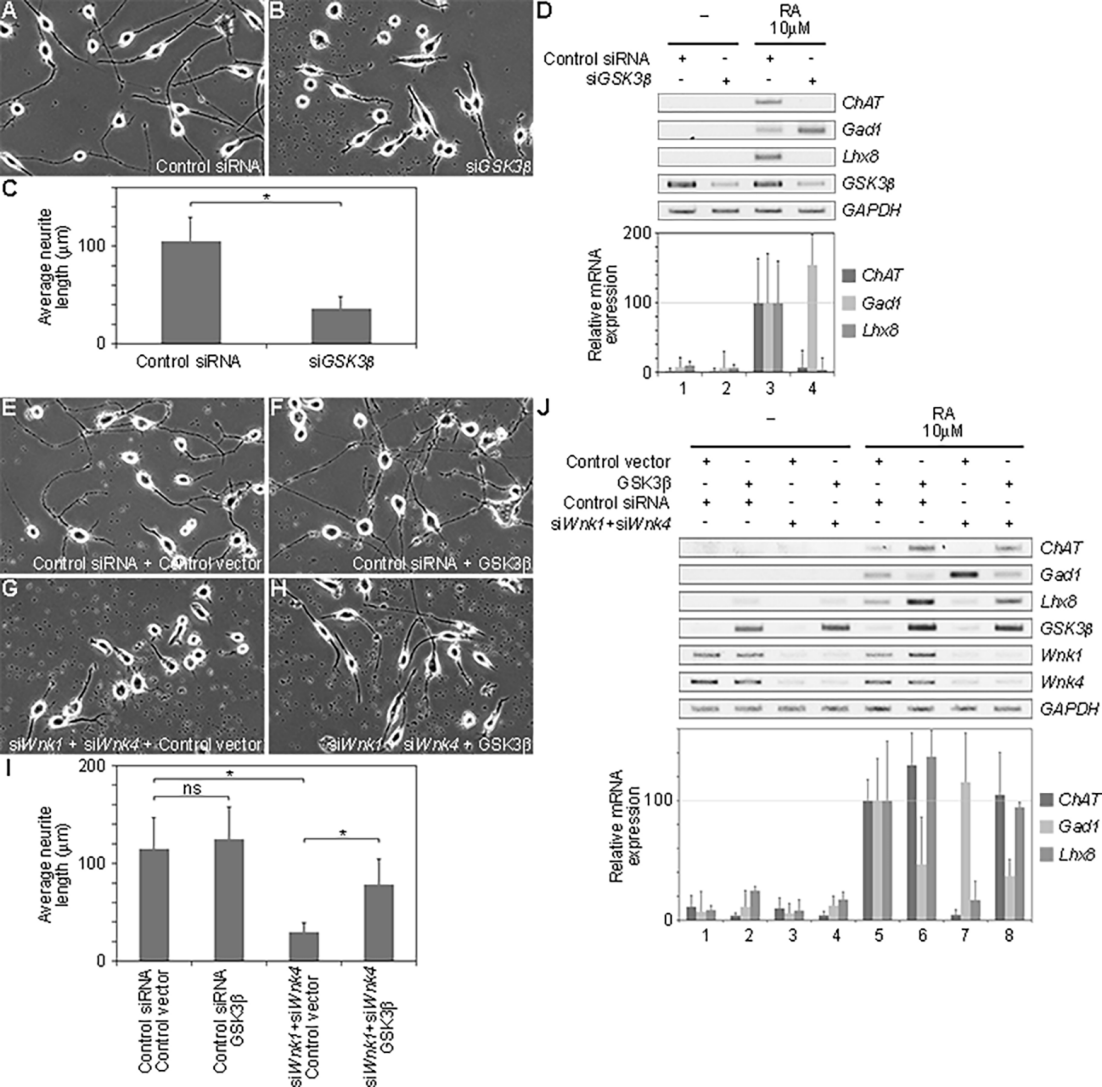
The WNK-OSR-GSK3ß pathway is involved in the neural development. (A–B) siRNA-treated differentiated Neuro2A cells induced by RA for 24 h; (A) Control siRNA, (B) si*GSK3ß*. (C) The average length of neurites in siRNA-treated differentiated Neuro2A cells induced by RA for 24 h, shown in A and B (Control siRNA (n = 93), si*GSK3ß* (n = 91)). * p<0.0005 calculated by the Student’s t-test. (D) Gene expression determined by RT-PCR or quantitative RT-PCR analysis was examined in Neuro2A cells. Cells treated with siRNA against *GSK3ß* (si*GSK3ß*); (lanes 1 and 2) undifferentiated cells, (lanes 3 and 4) cells differentiated by RA for 24 h. The value obtained from each sample was normalized to that of *GAPDH*. The value of *Lhx8*, *ChAT* or *Gad1* from differentiated cells under control siRNA treatment (lane 3) was set to 100. (E–H) Differentiated Neuro2A cells were transfected with various combinations of siRNAs and expression plasmids. (E) Control siRNA and control vector. (F) Control siRNA and GSK3ß. (G) si*Wnk1* and si*Wnk4*, and control vector. (H) si*Wnk1* and si*Wnk4*, and GSK3ß. (I) The average length of neurites in siRNA-treated differentiated Neuro2A cells induced by RA for 24 h, shown in E-H (Control siRNA and Control vector (n = 81), Control siRNA and GSK3ß (n = 87), si*Wnk1* and si*Wnk4*, and Control vector (n = 103), si*Wnk1* and si*Wnk4*, and GSK3ß (n = 77)). * p<0.0005 calculated by the Bonferroni correction. ns indicated non-significance. (J) Gene expression determined by RT-PCR or quantitative RT-PCR analysis was examined in Neuro2A cells. Cells were treated with various combinations of siRNAs and expression plasmids; (lanes 1–4) undifferentiated cells, (lanes 5–8) cells differentiated by RA for 24 h, (lanes 1–2 and 5–6) control siRNA, (lanes 3–4 and 7–8) si*Wnk1* and si*Wnk4*, (lanes 1, 3, 5 and 7) control vector, (lanes 2, 4, 6 and 8) GSK3ß. The value obtained from each sample was normalized to that of *GAPDH*. The value of *Lhx8*, *ChAT* or *Gad1* from differentiated cells under the treatment of control siRNA (lane 5) was set to 100.

As we showed previously [17], WNK plays an important role in neural specification through the regulation of *Lhx8* expression. We examined whether GSK3ß was also involved in neural specification downstream of WNK. Knockdown of *GSK3ß* caused the shortening of neurites after RA stimulation (Fig 4B compared with Fig 4A, quantified in Fig 4C). Knockdown of *GSK3ß* also decreased the expression of *Lhx8* and the choline acetyltransferase gene (*ChAT*; a marker for cholinergic neuron) (Fig 4D lane 4). However, the gene expression of glutamic acid decarboxylase 1 (*Gad1*; a marker for GABAergic neurons) increased (Fig 4D lane 4). These results suggest that GSK3ß is involved in neural specification, similar to that induced by the knockdown of both *Wnk1* and *Wnk4* as shown previously ([17]; see also Fig 4G and 4J).

We next examined whether the expression of GSK3ß suppressed the neural specification phenotypes caused by the knockdown of *Wnk*. While the expression of *GSK3ß* did not affect the elongation of neurites (Fig 4F compared with Fig 4E, quantified in Fig 4I), the expression of *GSK3ß* induced *Lhx8* and *ChAT* expression, and reduced *Gad1* expression after RA stimulation in Neuro2A cells (Fig 4J lanes 5 and 6). Under conditions of both *Wnk1* and *Wnk4* knockdown, the expression of *GSK3ß* partially rescued the elongation of neurites (Fig 4H compared with Fig 4G, summarized in Fig 4I), and *Lhx8* expression (Fig 4J lanes 7 and 8); this in turn increased *ChAT* expression and decreased *Gad1* expression (Fig 4J lanes 7 and 8). These results suggest that GSK3ß is involved in neural development and functions downstream of the WNK signaling pathway.

## Discussion

The WNK signaling pathway is involved in many biological processes, but the details of its components are unclear, except for in the kidney. Here, we screened candidate genes that genetically interact with the WNK signaling pathway in *Drosophila*. Among these, we identified *shaggy*, which encodes the *Drosophila* homolog of mammalian GSK3ß (Fig 1). We showed that GSK3ß activated *Lhx8* expression and that GSK3ß functions downstream of the WNK–OSR1 pathway by epistasis analysis (Fig 2). We also showed that GSK3ß might form a tertiary complex with WNK1 and OSR1 (Fig 3). Furthermore, GSK3ß was found to be involved in neural specification and neurite elongation (Fig 4), and GSK3ß rescued the neural phenotypes induced by the knockdown of both *Wnk1* and *Wnk4* (Fig 4). However, we did not observe direct phosphorylation of GSK3ß by WNK1 or OSR1 (Fig 3). This suggests that GSK3ß functions as a positive downstream effector in the WNK signaling pathway, although the regulation of GSK3ß activity by the signaling pathway remains unclear and requires further study to elucidate how WNK–OSR1 transduces the signal to GSK3ß.

GSK3ß plays many roles in various signaling pathways. In the PI3K/AKT signaling pathway, AKT phosphorylates Ser-9 of GSK3ß and inhibits GSK3ß activity for cell proliferation [22]. Previous research has shown that the PI3K/AKT signaling pathway activates the WNK–OSR1–NCC pathway to regulate blood pressure [20]. However, GSK3 was reported to be a negative regulator for the destruction complex in the Wnt signaling pathway [22]. A recent study showed that WNK is a positive regulator of the Wnt signaling pathway [18]. Here, we found that GSK3ß is a positive regulator downstream of the WNK–SPAK/OSR1 signaling pathway. These contradictions with regard to the regulation and role of GSK3ß clearly indicate that the WNK–SPAK/OSR1–GSK3ß signaling pathway for neural development is independent of PI3K/AKT or Wnt signaling pathways. The exact interaction between WNK and other signaling pathways remains to be determined and will require further analyses.

GSK3ß is also known to be involved in the Notch signaling pathway in which the intercellular domain (ICD) of Notch directly regulates the transcription of target genes with several co-factors [27]. GSK3ß was previously shown to bind and phosphorylate the Notch ICD which increased the transcriptional activity of the Notch ICD complex [28]. However, the mechanisms of GSK3ß activation in the Notch signaling pathway are still unclear. Since our initial screening of *Drosophila Wnk*-related genes showed that *Wnk* has a weak genetic interaction with the Notch signaling pathway (data not shown), we hypothesize that the WNK pathway positively regulates the Notch signaling pathway through GSK3ß in neural development. Further study will be required to prove this hypothesis, which is likely to be important for understanding the pathogenesis of HSAN2A.
